# The Clinical Efficacy and Safety of Nintedanib in the Treatment of Interstitial Lung Disease Among Patients With Systemic Sclerosis: Systematic Review

**DOI:** 10.1155/carj/1682546

**Published:** 2025-07-01

**Authors:** Khaled S. Al Oweidat, Ahmed A. Abdulelah, Ahmad A. Toubasi, Mohammad Abdulelah, Nour Z. Alatteili, Zaid A. Abdulelah

**Affiliations:** ^1^Department of Respiratory and Sleep Medicine, Department of Internal Medicine, The University of Jordan, Amman, Jordan; ^2^School of Medicine, The University of Jordan, Amman, Jordan; ^3^Faculty of Medicine, The University of Jordan, Amman, Jordan; ^4^Department of Internal Medicine, University of Massachusetts Chan Medical School—Baystate Campus, Springfield, Massachusetts, USA; ^5^Department of Cardiology, Royal Papworth Hospital, Cambridge, UK

**Keywords:** autoimmune lung disease, scleroderma, systemic sclerosis, tyrosine kinase inhibitor

## Abstract

Systemic sclerosis (SSc) is predominantly characterized by an array of cutaneous manifestations including Raynaud's phenomenon, calcinosis, telangiectasias, and skin fibrosis contributing toward substantial morbidity and diminished quality of life. The monumental impact of the disease regarding mortality is due to its pulmonary involvement known as SSc-associated interstitial lung disease (SSc–ILD). Currently, treatment is chiefly directed toward impeding disease progression with the mainstay treatment approaches involving the utilization of cyclophosphamide, mycophenolate mofetil, rituximab, and tocilizumab. Recently, a tyrosine kinase inhibitor, nintedanib, has been approved for the treatment of SSc–ILD and thus became the first medication to be fully licensed for SSc–ILD. A systematic review based on the Preferred Reporting Items of Systematic Review with Meta-analysis (PRISMA) was conducted after successful registration in PROSPERO to evaluate the efficacy and safety of nintedanib in SSc–ILD. We searched PubMed, Scopus, and CENTRAL up to the first of September 2023 utilizing the following keywords: ((Diffuse Parenchymal Lung Disease) OR (Diffuse Parenchymal Lung Diseases) OR (Interstitial Lung Disease) OR (Interstitial Lung Diseases) OR (Interstitial Pneumonia) OR (Interstitial Pneumonitis) OR (Pulmonary Fibrosis)) AND ((Systemic Scleroderma) OR (Systemic Scleroderma)) AND ((BIBF 1120) OR (BIBF-1120) OR (BIBF1120) OR (Nintedanib esylate) OR (Ofev) OR (Vargatef)). The clinical safety profile of nintedanib was deemed more favorable than other therapeutic regimens currently utilized, in addition to adequate clinical efficacy toward SSc–ILD.

## 1. Introduction

The collective interplay of vasculopathy, fibrosis due to dysregulated collagen production, and disruptive immunological pathways constitute the pathogenesis of the connective tissue disease systemic sclerosis (SSc) which is also commonly referred to as scleroderma [1]. SSc is predominantly characterized by an array of cutaneous manifestations including Raynaud's phenomenon, calcinosis, telangiectasias, and skin fibrosis contributing toward substantial morbidity and diminished quality of life. The monumental impact of the disease regarding mortality is due to its pulmonary involvement, principally the intricated pathophysiology involving alveolar epithelial damage with compounded profound inflammation, autoantibodies such as anti-topoisomerase I, and the interstitial thickening with the resultant interstitial lung disease (ILD), known as SSc-associated interstitial lung disease (SSc–ILD) that accounts for estimated SSc-related mortality approaching up to 35% [2–5].

Patients with SSc–ILD, more commonly in patients with diffuse SSc (dcSSc), typically present with fatigue, exertional dyspnea, and nonproductive cough. Nonetheless, the clinical course of SSc–ILD varies heterogeneously among patients, ranging from a chronic, indolent pattern to a progressively terminal nature [6]. SSc–ILD is characterized by a restrictive pattern on pulmonary function test (PFT) with a prominent reduction in forced vital capacity (FVC) and diffusing capacity for carbon monoxide (DLCO) [4]. Radiologically, the most frequently encountered pattern on high-resolution computed tomography (HRCT) is nonspecific interstitial pneumonia (NSIP), with other encountered patterns including interstitial pneumonic pattern, fluid-filled esophagus, and findings consistent with pulmonary hypertension such as right ventricular dilation [4, 7, 8]. Judiciously, efficacious and safe treatment approaches are mandated to address the clinical manifestations and complications associated with SSc–ILD. Currently, treatment is chiefly directed toward impeding disease progression with the mainstay treatment approaches that involve the utilization of cytotoxic alkylating agents, particularly cyclophosphamide (CYC), immunosuppressive agents with mycophenolate mofetil (MMF), and biologic agents such as anti-IL6 (tocilizumab) and anti-CD20 [rituximab (RTX)] [6].

However, the treatment approaches for SSc–ILD are continually evolving and progressing with new therapies emerging due to a greater understanding of the underlying pathophysiological pathways with one of the emerging medications for the treatment of SSc–ILD being nintedanib, a tyrosine kinase inhibitor that is targeted toward fibrotic mediators such as vascular endothelial growth factor receptor (VEGFR), platelet-derived growth factor (PDGF) receptor, and fibroblast growth factor receptor in addition to demonstrating profound inhibition of fibroblasts proliferation and myofibroblasts differentiation in in vitro studies [9, 10]. Due to the pharmacodynamics of nintedanib and the promising results of the 2019 SENSCIS trial, a randomized, double-blind, placebo-controlled clinical trial evaluating the use of nintedanib in SSc–ILD. With the primary endpoint assessing the annual rate of decline in FVC [11], nintedanib became the first FDA-approved treatment for SSc–ILD [4].

Accordingly, due to the paramount significance and the resultant morbidity and mortality associated with SSc–ILD and the dynamic nature of clinical research regarding clinical trials and drug discovery, we conducted a systematic review and meta-analysis of the currently existing literature on the utilization of nintedanib in the treatment of SSc–ILD to thoroughly evaluate its clinical efficacy and safety.

## 2. Material and Methods

### 2.1. Registration

We followed the Preferred Reporting Items of Systematic Review and Meta-analysis (PRISMA) in conducting this study. The protocol of this systematic review and meta-analysis was registered in PROSPERO, reference number, CRD42021269765, https://www.crd.york.ac.uk/prospero/display_record.php?RecordID=269765.

### 2.2. Search Strategy

PubMed, Scopus, and CENTRAL were searched up to the first of February 2023 utilizing the following keywords: ((Diffuse Parenchymal Lung Disease) OR (Diffuse Parenchymal Lung Diseases) OR (Interstitial Lung Disease) OR (Interstitial Lung Diseases) OR (Interstitial Pneumonia) OR (Interstitial Pneumonitis) OR (Pulmonary Fibrosis)) AND ((Systemic Scleroderma) OR (Systemic Scleroderma)) AND ((BIBF 1120) OR (BIBF-1120) OR (BIBF1120) OR (Nintedanib esylate) OR (Ofev) OR (Vargatef)). The search was done by A.T. and N.A. independently and any discrepancy was solved by discussion. The search results were imported to Rayyan (https://rayyan.ai/” https://rayyan.ai/). Duplicates were removed.

#### 2.2.1. Eligibility Criteria and Study Selection

Studies were included if they investigated the efficacy or safety of nintedanib on SSc–ILD on human or animal models. Studies were excluded if they were review articles or non-English articles. The study selection was done by screening the retrieved articles using title and abstract, and then, the remaining articles were tested against the inclusion criteria using their full-text form. The study selection was done by A.T. and N.A. independently, and any discrepancy was solved by discussion. The exposure of interest was nintedanib, while the outcome of interest was the drug efficacy, safety, and tolerability. The efficacy was assessed as (a) changes in “FVC (% of predicted)” from baseline; (b) changes in “DLCO (% of predicted)” from baseline; (c) mortality; (d) pulmonary artery systolic pressure; and (e) HRCT results change from baseline. Furthermore, additional efficacy outcomes were considered including changes from baseline in the modified Rodnan skin score, the total score on the St. George's Respiratory Questionnaire (SGRQ), the score on the Health Assessment Questionnaire–Disability Index (HAQ–DI), and the score on the Functional Assessment of Chronic Illness Therapy (FACIT)–Dyspnea questionnaire as well as changes in the cellular markers of the SSc disease progression. The safety was evaluated using (a) local side effects, (b) systemic side effects, and (c) unsolicited side effects. The tolerability was assessed by (a) treatment discontinuation, (b) treatment interruptions, (c) treatment dose reduction, and (d) treatment compliance.

#### 2.2.2. Data Extraction and Quality Assessment

The following variables were extracted from the included studies: study design, sample size, gender, age of the patients, and type of SSc as well as the efficacy, safety, and tolerability at baseline and after the treatment or for the treatment and placebo group according to the design of the study. The data extraction was done by A.T. and N.A. independently, and any discrepancy was solved by discussion. The quality assessment was done by A.A. and N.A. independently, and the discrepancy was solved by discussion. Regarding quality assessment tools, clinical trials were assessed using version 2 of Cochrane risk of bias assessment (ROB2) for randomized trials, and the cohort and case–control studies were assessed using Newcastle–Ottawa scale. For the animal studies, the Systemic Review Center for Laboratory Animal Experimentation (SYCRLE) ROB tool was utilized, while the Joanna Briggs Institute's (JBI) critical appraisal checklist for case reports was used for the only case report in the study.

#### 2.2.3. Summary Measures

The principal summary measures were the nintedanib efficacy, safety, and tolerability in the treatment of SSc-associated ILD. Data were narratively synthesized via characteristics, the outcomes, and the quality of the evidence of the included studies.

## 3. Results

### 3.1. Search Results

**Figure 1:**
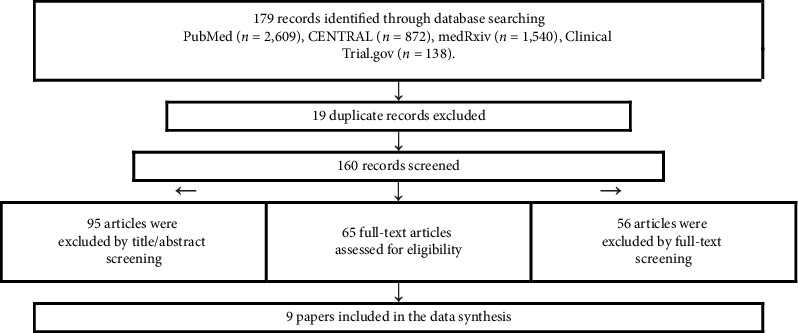
PRISMA flow diagram.

The search yielded 179 articles, of which 19 articles were duplicates. The remaining 160 articles were screened using title and abstract. As a result, 95 articles were excluded because they were reviews, commentaries, protocols, foreign languages, and no full text. The lasting 65 articles were screened using full-text form, and 9 articles were included.  Figure 1 shows the detailed process of study selection.

### 3.2. Study Characteristics

The general characteristics of the included studies are described in Table 1. A total of nine studies with 576 patients with ILD due to SSc and 11 animal models with SSc were included. Six articles were human studies [11–16], while three were animal studies [10, 17, 18]. One of the included studies by Distler et al. [11] was a randomized controlled clinical trial that compared the efficacy of nintedanib use as a treatment for SSc–ILD (SENSCIS trial). Another study by Seibold et al. [12] assessed the safety and tolerability of nintedanib use for SSc–ILD compared to placebo. Two of the included studies were subanalyses of the SENSCIS trial that were conducted by Kuwana et al. [13] and Azuma et al. [14] to assess the safety and efficacy of nintedanib use for SSc–ILD among the Japanese and Asian populations, respectively. Another study was a subanalysis of the SENSCIS trial that was conducted by Maher et al. [15]. The mean age for the nintedanib group was 53.7 ± 11.1, while it was 54.1 ± 12.7 for the placebo group. The results and outcomes summary of the included studies are summarized in Table 2. In terms of baseline therapies for the patients who were enrolled in the SENSICS clinical trial and thus its subanalysis studies [11–15], patients who were receiving prednisone at a dose of up to 10 mg per day or mycophenolate or methotrexate at a stable dose for at least 6 months before randomization (or both therapies) could participate in the trial. In instances where there was clinically significant progression of SSc–ILD, a number of additional therapies were considered for addition. These included MMF, mycophenolate sodium, methotrexate, azathioprine, CYC, cyclosporine A, prednisone > 10 mg/day, hydroxychloroquine, colchicine, D-penicillamine, sulfasalazine, RTX, tocilizumab, abatacept, leflunomide, tacrolimus, and newer antiarthritic treatments such as tofacitinib and potassium *para*-aminobenzoate. Nonetheless, there is no mention of how many patients required further therapies and which therapy was exactly introduced in such instances.

### 3.3. Quality of the Included Studies

Both randomized clinical trials by Distler et al. and Seibold et al. had a low overall ROB according to ROB2. The three subanalysis studies derived from the SENSCIS trial were not assessed as they have the same quality of the SENSCIS trial. The other human study conducted by Duarte et al. [16] had a low ROB in all JBI appraisal categories. On the other hand, none of the animal studies had a low ROB in all of the SYRCLE's ROB tool assessment categories. One of the animal studies by Atanelishvili et al. [17] had high ROB in concealing the animal allocation to different treatment group categories. Additionally, this study had high ROB in the random allocation of the animals to the treatment arms. The other animal study by Distler et al. had high ROB originating from the random selection for outcome assessment. The detailed ROB assessment of the included studies is described in Table 3.

### 3.4. Cellular Markers

Three studies observed the effect of nintedanib use on the cellular level (Table 1). Atanelishvili et al. showed that nintedanib was able to reduce the acute and the delayed increase of intracellular calcium entry induced by PDGF in a mice model of SSc–ILD. In addition, the study by Huang et al. showed that nintedanib was able to significantly inhibit the metabolic activity of PDGF-stimulated fibroblast. Furthermore, both studies showed that nintedanib reduced fibroblast proliferation and migration in a dose-dependent manner. The study by Atanelishvili et al. showed that nintedanib reduced PDGF and TGF beta-induced collagen Type 1 and fibronectin mRNA expression. Comparable results were reported by Huang et al. as their study showed that nintedanib reduced the mRNA expression of Col1a1, Col1a2, and fibronectin-1, as well as the release of collagen protein. Also, the same study showed that nintedanib was able to reduce the stimulatory effects of PDGF and TGFβ on myofibroblast differentiation and collagen release. Although Atanelishvili et al. reported that nintedanib had no effect on the basal levels of α-SMA, it was able to reduce PDGF-induced transcriptional activity of α-SMA in SSc lung fibroblasts. Likewise, nintedanib was able to reduce α-SMA expression induced by PDGF and TGFβ. On the other hand, Huang et al. showed that nintedanib decreased the mRNA levels of α-SMA at baseline and after the induction with PDGF. Furthermore, Atanelishvili et al. showed that nintedanib decreased the contractile activity of SSc–ILD lung fibroblast in a concentration-dependent manner. Huang et al. revealed that nintedanib reduced pulmonary fibrosis, hydroxyproline content, and myofibroblast counts. Similar results were reported by Huang et al. as nintedanib ameliorated the progression of fibrosis with significant decreases in dermal thickness, myofibroblast counts, and hydroxyproline content that was induced by bleomycin. Also, nintedanib induced regression in the pre-established fibrosis and reduced the dermal thickness, myofibroblast counts, and hydroxyproline content.

Overall, nintedanib proved to reduce the acute and the delayed increase of intracellular calcium entry induced by PDGF, inhibit the metabolic activity of PDGF-stimulated fibroblast, reduce fibroblast proliferation and migration, reduce pulmonary fibrosis, and decrease the contractile activity of SSc–ILD lung fibroblast. All of these cellular markers and pathways have been previously shown to be involved in the pathogenesis of SSc–ILD.

### 3.5. Lung Functions

#### 3.5.1. FVC

Five studies reported the FVC tests for their patients. The study conducted by Distler et al. showed that mean FVC at baseline was 72.4 ± 16.8 and 72.7 ± 16.6 for the treatment and control groups, respectively, whereas, at the end of the treatment, the mean decline in FVC was −1.4 ± 0.4 and −2.6 ± 0.4 for the treatment and the control groups, respectively. This difference in decline was statistically significant favoring the nintedanib group. Furthermore, this was interpreted as a significant difference in the annual rate of FVC decline in milliliter between the two groups favoring the nintedanib group (difference = 41.0; 95%CI: 2.9–79.0). Similarly, the annual rate of FVC percentage was significant between the groups favoring the nintedanib (difference = 1.2; 95%CI: 0.1–2.2). In addition, these findings were confirmed by the subanalysis conducted by Maher et al. as their study showed that nintedanib was significantly protective in the absolute decrease in FVC by 3.3% (OR = 0.68; 95%CI: 0.48–0.95) and significantly associated with the absolute increase in FVC by 3.3% (OR = 1.69; 95%CI: 1.11–2.59). Moreover, treatment with nintedanib was not significantly associated with an absolute decline of FVC of ≥ 5% predicted or death. Yet, it was significantly associated with the composite of an absolute decline in FVC of ≥ 10% predicted or death (HR = 0.64; 95% CI 0.43−0.95). On the other hand, the results from the subanalysis in the Japanese population showed that neither ≥ 3.0% absolute increase nor ≥ 3.3% absolute decrease was significantly associated with nintedanib. Likewise, the annual decline of FVC for the treatment group was not significantly different from the placebo group. Furthermore, the study conducted by Azuma et al. showed that the efficacy of nintedanib in reducing the rate of FVC decline was consistent between Asians and non-Asians.

#### 3.5.2. DLCO

Five studies reported DLCO tests for their patients. The study conducted by Distler et al. showed that mean DLCO at baseline was 52.9 ± 15.1 and 53.2 ± 15.1 for the treatment and control groups, respectively, whereas, at the end of the treatment, the mean decline in DLCO was −3.21 ± 0.54 and −2.77 ± 0.54 for the treatment and the control groups, respectively. These values of DLCO were interpreted as nonsignificant differences between the treatment and control groups. Moreover, the further analysis conducted by Maher et al. revealed that the treatment with nintedanib was significantly protected from an absolute decline of FVC of ≥ 10% predicted, an absolute decline in FVC of ≥ 5% to < 10% predicted with an absolute decline in DLCO of ≥ 15% predicted, or death (HR = 0.58; 95% CI: 0.39−0.87). Furthermore, in the case report done by Azuma et al., the patient after using nintedanib showed improvement in FVC% from 40.5% to 52.1%, while the DLCO was stable throughout the duration of the study at 15%.

### 3.6. Pulmonary Artery Hypertension and Fibrosis

Two studies reported the effect of nintedanib treatment on pulmonary artery outcomes. The study by Seibold et al. showed that there was no difference in the pulmonary pressure between the nintedanib and placebo groups. In comparison, the study conducted by Huang et al. showed that nintedanib significantly reduced the thickening of the walls of pulmonary arteries and decreased the number of blocked vessels. Similarly, the nintedanib reduced the number of proliferating vascular smooth muscle cells. Also, nintedanib inhibited the proliferation of human pulmonary vascular smooth muscle cells in vitro, both under normal conditions and on stimulation with PDGF.

### 3.7. Mortality

One study reported the mortality among its patients. The study by Distler et al. showed that nintedanib use was significantly associated with improvement in the mortality outcomes among patients with ILD due to SSc.

### 3.8. Subjective Outcomes

#### 3.8.1. St. George's Respiratory Questionnaire

Two studies reported the outcomes in terms of St. George's Respiratory Questionnaire. Distler et al. showed that there was no significant difference between the treatment and the placebo groups. Likewise, the same findings were found in the subanalysis for the Japanese population conducted by Azuma et al.

#### 3.8.2. Score on the HAQ–DI

One study reported the score of HAQ–DI for its patients. Distler et al. revealed that there was no significant difference between the nintedanib and placebo groups in HAQ–DI scores at the end of follow-up.

#### 3.8.3. Modified Rodnan Skin Score

Three studies reported the outcomes in terms of modified Rodnan skin score. The study by Distler et al. showed that there was no significant difference between the treatment and the placebo groups. Similarly, these findings were supported in the subanalysis for the Japanese population and the Asian population conducted by Kuwana et al. and Azuma et al., respectively.

#### 3.8.4. FACIT

One study reported the scores of FACIT for its patients. Distler et al. demonstrated that there was no significant difference in the FACIT–dyspnea scores between the nintedanib and placebo groups.

### 3.9. Adverse Effects

#### 3.9.1. Systemic Side Effects

Systemic side effects were reported by four of the included articles (Table 2). The study by Distler et al. showed that diarrhea occurred in 75.7% of the nintedanib group, while it occurred only in 31.6% in the placebo group. The subanalysis conducted by Kuwana et al. among the Japanese population showed that diarrhea occurred more frequently among them than the general population at a rate of 82.4%. Similarly, the study by Azuma et al. showed that among the Asian populations, diarrhea occurred more frequently than the general population at a rate of 80.6%. The study by Distler et al. showed that nausea and vomiting occurred in 31.6% and 24.7%, while it occurred in 13.4% and 10.4% in the placebo group, respectively. Lower rates of nausea and vomiting were reported by Azuma et al. and Kuwana et al. among the Asian and Japanese populations, respectively. In comparison, Distler et al. and Azuma et al. showed that abdominal pain occurred at similar rates among the nintedanib and the placebo groups. Weight reduction was reported by Distler et al. at a rate of 11.8% among the nintedanib group, while the rate was 4.2% in the placebo group. Similar rates of weight reduction were reported in the subanalysis conducted among the Japanese and the Asian populations separately. Distler et al. showed that skin ulcers occurred at similar rates between the nintedanib and placebo groups at a rate of 18.4% and 17.4%, respectively. On the other hand, skin ulcers occurred more frequently in the Japanese and Asian populations as reported in the two subanalyses. The studies by Distler et al. and Azuma et al. showed that cough occurred less frequently in the nintedanib group compared to the placebo group. Furthermore, the studies by Distler et al. and Azuma et al. reported that nasopharyngitis occurred less frequently in the nintedanib group compared to the placebo group. Differently, Kuwana et al. showed that nasopharyngitis in the subanalysis of the Japanese population occurred more frequently in the nintedanib group compared to the placebo one. Moreover, Distler et al. and Azuma et al. reported that the upper respiratory tract infections occurred at a similar frequency among the nintedanib and placebo groups. Distler et al. showed that fatigue occurred more in the nintedanib group compared to the placebo group, while it occurred at a similar frequency in the placebo and nintedanib groups in the Asian population subanalysis conducted by Azuma et al. Distler et al. reported that liver enzymes three times the normal occurred more frequently in the nintedanib group (4.9%) compared to placebo groups (0.7%). Similarly, the subanalysis in the Japanese population showed that liver test abnormalities were reported in higher frequency in the nintedanib group compared to the placebo groups. In comparison, it occurred at similar rates among the nintedanib and placebo groups in the subanalysis of the Japanese population conducted by Azuma et al. The study by Seibold et al. showed that among the nintedanib group events occurred in this frequently 11.1% bleeding, 2.8% epistaxis, 2.4% contusions, 1.4% serious bleeding events, 4.9% hypertension, 1% liver enzymes five times the normal, 0% liver enzymes eight times the normal, 0.3% bilirubin 1.5 and 3 times the normal, 3.5% ALP 1.5 times the normal, and 1% ALP 2 times the normal. On the other hand, the study showed that among the placebo group events occurred in this frequently 8.3% bleeding, 3.8% epistaxis, 1.0% contusion, 0.7% serious bleeding events, 1.7% hypertension, 0.3% liver enzymes five times the normal, 0.3% liver enzymes eight times the normal, 0% bilirubin 1.5 and 3 times the normal, 1% ALP 1.5 times the normal, and 0% ALP 2 times the normal.

Further information regarding severe adverse events, serious adverse events, fatal adverse events, tolerability, treatment discontinuation, dose reduction, treatment interruptions, compliance, and adverse events leading to permanent termination is highlighted in Table 2.

## 4. Discussion

Despite the detrimental outcomes and the significant morbidity and mortality associated with SSc–ILD, there remains a considerable lack of a substantially effective treatment with the currently utilized treatment regimens being limited to MMF, CYC, and RTX. Nonetheless, with the emergence of nintedanib, we conducted this vigorous critical assessment of the currently available studies to determine its clinical efficacy and safety in patients with SSc–ILD to establish its role in the treatment of SSc–ILD in comparison with the currently utilized immunotherapies. The utilization of nintedanib as a therapy for SSc–ILD is based on its pharmacodynamics as it targets several fibrotic mediators and thus exhibits a multitude of therapeutic benefits such as antifibrotic and anti-inflammatory action with the typical treatment protocol involving oral administration of nintedanib 150 mg twice daily based on the findings of the SENSCIS trial [9, 12].

Nintedanib demonstrates its intracellular inhibition of tyrosine kinases through inhibition of PDGF which consequently impact the normally increasing level of intracellular calcium, prevent the stimulatory effects that PDGF exhibits on myofibroblast differentiation and collagen release, and reduce PDGF-induced transcriptional activity of α-SMA in SSc lung fibroblasts [17]. Additionally, the arrest of progression of SSc–ILD that occurs with the utilization of nintedanib is postulated to occur on the cellular and molecular level due to its ability to reduce pulmonary fibrosis; in addition to inducing the regression of the pre-established fibrosis, reduce myofibroblast counts and hydroxyproline content that drive the progressive fibrosis known to occur in SSc–ILD [10, 18]. Accordingly, the aforementioned cellular pathways that are impacted by nintedanib result in the therapeutic benefit witnessed on the clinical level.

On the clinical level, the predominant therapeutic effect of nintedanib on the FVC was determined to be significantly superior to the control groups as both the difference in the annual rate of FVC decline in milliliter (difference = 41.0; 95%CI: 2.9–79.0) and the annual rate of FVC percentage (difference = 1.2; 95%CI: 0.1–2.2) favored the nintedanib group [11]. Furthermore, nintedanib was proven to be significantly protective in the absolute decrease in FVC by 3.3% (OR = 0.68; 95%CI: 0.48–0.95) and significantly associated with the absolute increase in FVC by 3.3% (OR = 1.69; 95%CI: 1.11–2.59) [15]. Furthermore, nintedanib did not demonstrate efficacy on the DLCO as the mean decline was determined to be nonsignificant when the treatment group was compared with the control group [11]. Accordingly, the effect of nintedanib appears to be limited toward FVC only. In comparison with other treatment regiments utilized in SSc–ILD, Sircar et al. [19] conducted a systematic review with meta-analysis to evaluate the efficacy of RTX in comparison with MMF and CYC in patients with SSc–ILD and demonstrated that RTX is superior in terms of improving FVC in the first 6 months of treatment in comparison with other immunotherapies as the FVC improved by 1.03% (95%CI: 0.11–1.94), while the improvement in FVC was similar to control groups at the 12-month interval. Additionally, Sircar et al. [19] assessed the studies where RTX was evaluated solely where the FVC was proven to be improved by 4.49% (95%CI:0.25–8.73) at the 6-month interval and by 7.03% (95%CI:4.37–9.7) at the 12-month interval, while in regard to the effects of RTX on the DLCO, it was demonstrated that RTX improved the DLCO parameter by 3.47% (95%CI:0.99–5.96) at the 6-month interval and by 4.08% (95%CI: 1.51–6.65) at 12 months, but through the conduction of sensitivity analysis, the effects on DLCO were determined to be precarious. Accordingly, when comparing nintedanib and RTX, nintedanib does hinder the accelerated progression of SSc–ILD through decelerating the decline in FVC, while RTX demonstrates its superiority in improving the FVC, both at 6 months and 12 months from the initiation of treatment, thus indicating potential therapeutic benefit from the combined use of both regimens. Nevertheless, further studies evaluating treatment protocols consisting of combined nintedanib and RTX are warranted to establish their combined therapeutic benefits on the FVC and DLCO in SSc–ILD. Nintedanib however did not demonstrate any significant impact on any of the several scores utilized for the assessment of SSc–ILD when compared to the control groups [11, 13, 14]. Nonetheless, other forms of therapies, particularly RTX, did exhibit a significant decrease in the modified Rodnan skin score potentially due to the systemic action of RTX.

Nintedanib induces gastrointestinal manifestations, most commonly diarrhea, in a significant portion of the patients reaching as high as 75.7%, with higher incidence in Asian populations. Other commonly reported gastrointestinal manifestations included nausea and vomiting occurring in 31.6% and 24.7%, respectively. The gastrointestinal manifestations due to nintedanib were investigated in both idiopathic pulmonary fibrosis and SSc–ILD, with findings demonstrating similar risk factors to developing these manifestations in both diseases [12]. Patients with poor performance status, high body mass index (BMI), high modified Medical Research Council Dyspnea score, and high GAP index are more likely to develop nausea, while high GAP index and/or low BMI were associated with higher likelihood of diarrhea [20]. Surprisingly however, the effect of nintedanib in decelerating the prominent decline in FVC was unaffected by dosage adjustments done to reduce the severity and frequency of the adverse events and thus can significantly improve compliance and adherence to the treatment, which is documented to be at around 95.5%, especially since most of the treatment discontinuations (16%) are attributed to gastrointestinal manifestations, notably diarrhea [12]. One the other hand, fatal adverse events were approximately similar between the nintedanib and control groups and thus indicating the absence of any significant and alarming life-threatening events. Despite these reported serious and fatal adverse events, nintedanib demonstrated improved outcomes, notably in mortality outcomes, in patients with SSc–ILD when compared to placebo groups with that being of crucial importance as it significantly and positively impacts the benefit–risk ratio.

In comparison with the other utilized therapies in SSc–ILD, CYC appears to induce adverse events in 70% of patients in one particular study [19], and treatment with CYC is typically discontinued prematurely due to its adverse events. While RTX appears to be safer than CYC and MMF as it results in the less infectious adverse events in a population of patients who are at increased risk of recurrent respiratory infections [19, 21], nintedanib did not demonstrate any prominent concerns of infectious adverse events in the SENSICS trial [11]. However, bleeding events are of particular concern in nintedanib in contrast to the other treatment regimens due to the inhibitory effects of nintedanib on VEGFR which consequently results in the increased risk of bleeding [22].

Arguably, the number of included articles in this systematic review potentially limits the generalization of the evidence provided regarding the clinical efficacy and safety of nintedanib. Additionally, the included animal studies demonstrated the presence of high ROB, and accordingly, their findings might potentially hinder the currently available evidence on the efficacy and safety of nintedanib. The inclusion of only English published articles also limits the generalizability of our findings. Furthermore, the difference in the efficacy and safety outcomes assessment between the included studies limited our ability to conduct a meta-analysis for the included studies. Thus, future randomized controlled trials are recommended to use similar methods in assessing their outcomes. Nonetheless, we strongly believe that this study further validates the benefits of utilizing nintedanib in the treatment of SSc–ILD.

## 5. Conclusion

In conclusion, this study demonstrates that nintedanib is safe and effective in the treatment of SSc–ILD, but the effect of this agent might be augmented by using additional treatment regimens. Accordingly, we await more data from randomized controlled trials to confirm our findings regarding the safety and efficacy of nintedanib and to evaluate the efficacy and safety of using nintedanib with other disease-modifying agents in the management of SSc–ILD.

## Figures and Tables

**Table 1:** General characteristics of included studies.

Study	Country	Design	Sample size	Patients on nintedanib	Patients on placebo	Age of nintedanib patients (mean ± SD)	Age of placebo patients (mean ± SD)
Distler et al.	Multinational	RCT	576	277	293	53.4 ± 12.6	53.4 ± 12.6
Seibold et al.	Multinational	RCT (subanalysis)	576	277	293	53.4 ± 12.6	53.4 ± 12.6
Maher et al.	Multinational	RCT (subanalysis)	576	277	293	53.4 ± 12.6	53.4 ± 12.6
Atanelishvili et al.	—	Animal study	—	—	—	—	—
Huang et al.	Germany	Animal study	—	—	—	—	—
Duarte et al.	Portugal	Case report	1	—	—	—	—
Kuwana et al.	Japan	RCT	70	36	34	52.0 ± 7.4	52.3 ± 12.6
Azuma et al.	Japan	RCT	143	62	81	55.2 ± 13.1	51.8 ± 13.3

**Table 2:** Summary of outcomes of the included study findings.

**Study**	**FVC**				
	**Variable**	**Control**		**Treatment**	
		**Baseline**	**Follow-up**	**Baseline**	**Follow-up**

Distler et al.	FVC	72.7 ± 16.6	70.1 ± 16.2	72.4 ± 16.8	71.1 ± 16.4
	Annual FVC decline (mm)	MD = 41.0; 95% CI: 2.9–79.0			
	Annual FVC decline (%)	MD = 1.2; 95% CI: 0.1–2.2			
Maher et al.	Absolute decrease in FVC by 3.3%	OR = 0.68; 95% CI: 0.48–0.95			
	Absolute increase in FVC by 3.3%	OR = 1.69; 95% CI: 1.11–2.59			
	Absolute decline of FVC of ≥ 10% predicted or death	HR = 0.64; 95% CI 0.43−0.95			

**Study**	**DLCO**				
	**Variable**	**Control**		**Treatment**	
		**Baseline**	**Follow-up**	**Baseline**	**Follow-up**

Distler et al.	DLCO	53.2 ± 15.1	50.43 ± 14.6	52.9 ± 15.1	49.7 ± 14.10
Maher et al.	Absolute decline of FVC of ≥ 10%, decline in FVC of ≥ 5% to < 10% with an absolute decline in DLCO of ≥ 15%, or death	HR = 0.58; 95% CI: 0.39−0.87			
Azuma et al.	DLCO	—	—	15%	15%

**Study**	**Systematic side effects**				
	**Variable**	**Control (%)**	**Treatment (%)**		

Distler et al.	Diarrhea	31.6	75.7		
Kuwana et al.	Diarrhea	—	82.4		
Azuma et al.	Diarrhea	—	80.6		
Distler et al.	Nausea	13.4	31.6		
Distler et al.	Vomiting	10.4	24.7		
Distler et al.	Weight reduction	4.2	11.8		
Distler et al.	Skin ulcer	17.4	18.4		
Distler et al.	Liver enzymes ∗3 the normal	0.7	4.9		

**Study**	**Other side effects**				
	**Variable**	**Control (%)**	**Treatment (%)**		

Distler et al.	Severe side effects	12.5	18.1		
Distler et al.	Serious side effects	21.5	24.0		
Kuwana et al.	Serious side effects	—	25.8		
Azuma et al.	Serious side effects	—	26.5		
Distler et al.	Fatal adverse effects	1.4	1.7		
Azuma et al.	Fatal adverse effects	1.2	1.6		

**Study**	**Tolerability**				
	**Variable**	**Control (%)**	**Treatment (%)**		

Distler et al.	Treatment discontinuation	8.7	16.0		
Kuwana et al.	Treatment discontinuation	5.6	17.6		
Azuma et al.	Treatment discontinuation	8.6	19.4		
Seibold et al.	Dose reduction	4.5	36.1		
Seibold et al.	Treatment interruptions	11.5	37.8		
Seibold et al.	Compliance	96.4	95.5		
Seibold et al.	Permanent termination	8.7	16.0		

*Note:* DLCO = diffusing capacity for carbon monoxide.

Abbreviations: FEV = fractional expiratory volume, FVC = fractional vital capacity.

**Table 3:** Quality assessment of the included studies.

Study	Randomization	Deviation from intervention	Missing data	Outcome measurement	Selection of outcome reporting	Selection of results	Overall risk
Distler et al.	Low	Low	Low	Low	Low	Low	Low
Seibold et al.	Low	Low	Low	Low	Low	Low	Low
Maher et al.	Low	Low	Low	Low	Low	Low	Low
Kuwana et al.	Low	Low	Low	Low	Low	Low	Low
Azuma et al.	Low	Low	Low	Low	Low	Low	Low
Duarte et al.	Low	Low	Low	Low	Low	Low	Low
Atanelishvili et al.	Unclear	High	High	Unclear	Unclear	Unclear	Low
Huang et al. (2017)	Unclear	Unclear	Unclear	High	Unclear	Low	Low
Huang et al.	Unclear	Low	Unclear	Unclear	Unclear	Low	Low

## Data Availability

All the data presented in this study are retrieved from the respective published scientific works as referenced. No other data are available.
